# Sinovac COVID-19 Side Effects in Hypertensive Patients: An Observational Study From Pakistan

**DOI:** 10.7759/cureus.40444

**Published:** 2023-06-15

**Authors:** Ahsan Masood, Hira Khalid Chohan, Muhammad Mubeen, Muhammad Faizan, Subhana Moin, Musarat Khalid Chohan, Tatheer Syed, Adnan Anwar, Atif A Hashmi

**Affiliations:** 1 Internal Medicine, Gomel State Medical University, Gomel, BLR; 2 General Surgery, Dow University of Health Sciences, Karachi, PAK; 3 Internal Medicine, Dow University of Health Sciences, Karachi, PAK; 4 Public Health Sciences, Health Department of Sindh, Karachi, PAK; 5 Dentistry, Hamdard College of Medicine and Dentistry, Karachi, PAK; 6 Anaesthesiology, Jinnah Sindh Medical University, Karachi, PAK; 7 Public Health Sciences, Jinnah Sindh Medical University, Karachi, PAK; 8 Physiology, Hamdard College of Medicine and Dentistry, Karachi, PAK; 9 Internal Medicine, Essa General Hospital, Karachi, PAK; 10 Pathology, Liaquat National Hospital and Medical College, Karachi, PAK

**Keywords:** covid-19, vaccine, swelling, pain, fever, hypertension, sinovac vaccine

## Abstract

Background

The most important factor in combating the coronavirus disease 2019 (COVID-19) pandemic was the provision of safe and effective vaccines. The acceptance of vaccines is impacted by several variables, including beliefs about the vaccine’s safety and adverse effects. Vaccine side effects can vary depending on the type, but they are often moderate, localized, transient, and self-limiting. Therefore, this study aimed to assess the prevalence of side effects experienced after receiving the Sinovac vaccine by participants hypertensive and non-hypertensive participants.

Methodology

This was a cross-sectional, multicenter study that was performed using non-probability sampling. The study duration was six months from May 1, 2022, to October 31, 2022. The study involved 600 individuals who had either received the first or second dose of the Sinovac vaccine. For categorical data, frequencies and percentages were documented. The chi-square test was applied to determine the association between local and systemic side effects among hypertensive and non-hypertensive participants.

Results

The study findings showed that out of 600 participants, there were 187 (62.3%) males and 113 (37.7%) females with hypertension, and 222 (74.0%) males and 78 (26.0%) females without hypertension, with a significant association (p = 0.002). Following the first dose of the Sinovac vaccine, fever was the most commonly reported side effect in 153 (51.0%) hypertensive participants and 62 (20.7%) non-hypertensive participants, with a significant association (p < 0.001). Similarly, following the second dose of the Sinovac vaccine, fever was the most commonly reported side effect in 108 (36.0%) hypertensive participants and 57 (19.0%) non-hypertensive participants, with a significant association (p < 0.001).

Conclusions

This study concluded that the presence of hypertension significantly increased the manifestations of local and systemic side effects compared with non-hypertensive participants. Moreover, fever, pain, and swelling at the injection site were the most commonly reported side effects after receiving the first and second doses of the Sinovac vaccine.

## Introduction

In December 2019, the severe acute respiratory syndrome coronavirus 2 (SARS-CoV-2) pandemic, which had its beginnings in Wuhan, China, sparked a catastrophic worldwide crisis. There is currently no recognized cure for coronavirus disease 2019 (COVID-19). A vaccine was imperative for controlling the pandemic. Vaccinations continue to avert millions of deaths annually around the world through either total immunity from the disease or relief from its symptoms. On March 16, 2020, the first COVID-19 vaccine candidate quickly began clinical testing [1]. On January 14th, 2021, Turkey approved the emergency use of the CoronaVac vaccination for healthcare professionals, the elderly (age above 65), and people with comorbidities. Initially, two dosages were administered, separated by four-week intervals [2].

According to the World Health Organization (WHO) and health professionals, therapeutic medications are less safe than vaccines [3]. However, side effects associated with vaccines may arise due to the pharmacokinetics as well as the toxicity of vaccines, interactions with additional medicines, and hypersensitivity reactions (HRs). Although allergic and non-allergic mechanisms cause HRs, immunoglobulin (Ig)-E-mediated allergic reactions are the most common type [4]. Although vaccine-related anaphylaxis, which often develops within 15 to 30 minutes and can be fatal, is the most severe HR, it is treatable with adrenalin without side effects [5].

Several COVID-19 vaccines that are currently used worldwide include inactivated vaccines (Sinopharm, Sinovac, and COVAXIN), viral vector vaccines (AstraZeneca, Sputnik V), and mRNA vaccines (Pfizer BioNTech, Moderna, and Johnson & Johnson) [6]. Each individual involved in the process, including the person receiving the vaccine, caretakers, and healthcare professionals, must be aware of any potential adverse effects [7].

One of the most frequently used vaccinations in many nations is the Sinovac vaccine, which is a Chinese inactivated virus vaccine. The Sinovac and Pfizer vaccines had efficacy rates of 83.5% and 95%, respectively [8,9]. The Sinovac vaccine is one of the highly administered COVID-19 vaccines, and its potential adverse consequences have been studied. According to various clinical trials of the COVID-19 vaccine, high temperature or allergic responses that include itchiness and inflammatory conditions were among the less serious adverse events triggered by COVID-19 vaccines. In contrast, pain and injection site swelling were frequently detected side effects with a speedy recovery within 48 hours after vaccination [10-12]. The most frequent side effect related to practically all COVID-19 vaccinations was pain at the injection site. Other modest side effects of the Sinovac vaccine included weakness, muscle aches, and diarrhea, which were mild in intensity and continued only for two days. Contrary to other COVID-19 vaccines, CoronaVac/Sinovac recipients experienced fewer fever episodes. The Sinovac vaccine was advised for people 18 years of age and above [12].

A significant public health concern is vaccine hesitancy, which is fostered by misconceptions and false information regarding the efficacy and safety of vaccines [13]. Knowledge of the vaccine, beliefs about its side effects, perceptions of vaccination, perceived susceptibility to sickness, social consequences, and increased vaccine information are just a few of the variables that can affect vaccine acceptance [14]. According to a large-scale international survey, healthcare workers from the western regions of the Arab world (Egypt, Tunisia, Morocco, and Algeria) had the highest rates of vaccination reluctance. The most frequently mentioned reason for reluctance was concerns about side effects [15].

Throughout the epidemic, vaccine uptake and reluctance fluctuated and were dynamic, with COVID-19 having the most impact [16]. Immunization resistance was listed by the WHO as one of the top 10 dangers to global health in 2019 even before the epidemic [17]. Consequently, the COVID-19 pandemic made this problem much more complex. The global population exhibited resistance to vaccines despite the fact that vaccinations were essential for everyone. The hesitation to get vaccinated was brought on by skepticism in research analysis and the vaccines, as well as concerns over the vaccines’ rapid development, harmful side effects, and other adverse reactions [18]. One of the concerns that had an impact on public health compliance was anxiety [19]. To better limit the spread of COVID-19, measures were developed to increase the individual acceptability of COVID-19 immunization. It is important to determine the causes and influences that can boost vaccination acceptability, particularly among workers who are more susceptible to COVID-19.

Although the Sinovac COVID-19 vaccine is often used in several countries, there is a paucity of published data to support any adverse effects. Therefore, this research aimed to ascertain if participants with and without hypertension experienced any of the reported side effects of the Sinovac vaccine.

## Materials and methods

This was a multicenter, prospective, cross-sectional study that was performed using non-probability sampling. The study duration was six months from May 1, 2022, to October 31, 2022. The study was approved by Essa General Hospital (Essa/25/2023). The study involved 600 individuals who had either received the first or second dose of the Sinovac vaccine. Vaccine participants of both genders above 18 years of age were included in the study. Participants with active COVID-19 or any other active infection were excluded from the study. Similarly, participants with advanced renal or liver diseases and those undergoing chemoradiation for malignancies were excluded from the study. Participants who had previously received a COVID-19 immunization, had never received one, or had received a different vaccine rather than Sinovac were excluded from the study.

Each participant was asked for their informed consent and given a brief explanation of the study goals. Participant data were collected using a predesigned questionnaire. Demographic information on the participants included their gender, age, comorbidities, Sinovac vaccination with both dosages, previous exposure to COVID-19 infection, and the prevalence of any local and systemic side effects after receiving the first and second doses of the vaccine. The following symptoms were deemed as systemic side effects: fever, chills, headache, dyspnea, nausea, diarrhea, joint pain, lymphadenopathy, painful throat, tension, and fatigue. The injection site may experience discomfort, burning, redness, and swelling as local side effects. Participant satisfaction was also noted.

Data analysis

The data were analyzed using SPSS Statistics for Windows, Version 26.0 (IBM Corp., Armonk, NY, USA). Frequencies and percentages were documented for categorical data such as gender, comorbidities, previous COVID-19 infection, and post-vaccination side effects. Continuous data, for instance, age, height, weight, and duration of comorbidities, were presented as means and standard deviations. The chi-square test was applied to determine the association between local and systemic side effects among hypertensive and non-hypertensive participants. A p-value <0.05 was considered statistically significant.

## Results

A total of 600 recipients who were completely vaccinated with the Sinovac vaccine were studied. There were 187 (62.3%) males and 113 (37.7%) females with hypertension, and 222 (74.0%) males and 78 (26.0%) females did not have hypertension, with a significant association among them (p = 0.002). The mean age of the hypertensive participants was 43.42 ± 14.33 years, and the mean age of the non-hypertensive participants was 42.52 ± 13.32 years, with an insignificant association among them (p = 0.428). The mean weight of the hypertensive participants was 65.94 ± 14.50 kg and that of the non-hypertensive participants was 65.66 ± 13.16 kg, with an insignificant association among them (p = 0.809). The mean height of the hypertensive participants was 5.26 ± 0.73 feet and that of the non-hypertensive participants was 5.37 ± 0.60 feet, with a slightly significant relationship among them (p = 0.054). The mean duration of hypertension was 4.85 ± 4.11 years. The mean duration of diabetes was 5.09 ± 3.72 years in hypertensive participants and 7.57 ± 3.89 years in non-hypertensive participants, with a significant association among them (p = 0.003). Out of 600 participants, only 44 (14.7%) and 42 (14.0%) had diabetes in hypertensive and non-hypertensive participants, respectively, with an insignificant association among them (p = 0.816). Additionally, only nine (3.0%) hypertensive and three (1.0%) non-hypertensive participants had past exposure to COVID-19 infection, with an insignificant difference noticed among them (p = 0.08), as presented in Table 1.

**Table 1:** Demographic details of participants vaccinated by Sinovac vaccine (n=600) P-value significant at <0.05.

Variables		Values		p-value
		Hypertensive participants	Non-hypertensive participants	
Age (years), mean ± SD		43.42 ± 14.33	42.52 ± 13.32	0.428
Weight (kg), mean ± SD		65.94 ± 14.50	65.66 ± 13.16	0.809
Height (ft), mean ± SD		5.26 ± 0.73	5.37 ± 0.60	0.054
Diabetes mellitus duration (years), mean ± SD		5.09 ± 3.72	7.57 ± 3.89	0.003*
Gender, n (%)	Male	187 (62.3%)	222 (74.0%)	0.002*
	Female	113 (37.7%)	78 (26.0%)	
Diabetes mellitus, n (%)	Yes	44 (14.7%)	42 (14.0%)	0.816
	No	256 (85.3%)	258 (86.0%)	
Previously infected with COVID-19, n (%)	Yes	9 (3.0%)	3 (1.0%)	0.080
	No	291 (97.0%)	297 (99.0%)	

Following the first dose of the Sinovac vaccine, fever was the most frequently reported side effect in 153 (51.0%) hypertensive participants and 62 (20.7%) non-hypertensive participants, with a significant association noticed among them (p < 0.001). Additionally, injection site pain was found in 91 (30.3%) hypertensive participants and 73 (24.3%) non-hypertensive participants, with an insignificant association found among them (p = 0.099). Moreover, other side effects such as burning, swelling, and redness at the site of injection; chills; headaches; rashes; fatigue; joint pain; muscle ache; and chest pain in hypertensive and non-hypertensive participants were significantly associated (p < 0.05). On the other hand, nausea, diarrhea, and cough were the least reported side effects in hypertensive and non-hypertensive participants, with a significant relationship observed among them (p < 0.05), as presented in Table 2.

**Table 2:** The prevalence of side effects after receiving the first dose of the Sinovac vaccine among hypertensive and non-hypertensive participants. P-value significant at <0.05.

Variables		Hypertensive participants, n (%)	Non-hypertensive participants, n (%)	P-value
Pain at the site of injection	Yes	91 (30.3%)	73 (24.3%)	0.099
	No	209 (69.7%)	227 (75.7%)	
Swelling at the site of injection	Yes	93 (31.0%)	65 (21.7%)	0.009*
	No	207 (69.0%)	235 (78.3%)	
Redness at the site of injection	Yes	48 (16.0%)	24 (8.0%)	0.003*
	No	252 (84.0%)	276 (92.0%)	
Lymphadenopathy	Yes	36 (12.0%)	6 (2.0%)	<0.001*
	No	264 (88.0%)	294 (98.0%)	
Fever (temperature >37.8˚C)	Yes	153 (51.0%)	62 (20.7%)	<0.001*
	No	147 (49.0%)	238 (79.3%)	
Headache	Yes	46 (15.3%)	14 (4.7%)	<0.001*
	No	254 (84.7%)	286 (95.3%)	
Nausea	Yes	33 (11.0%)	9 (3.0%)	<0.001*
	No	267 (89.0%)	291 (97.0%)	
Rashes	Yes	57 (19.0%)	27 (9.0%)	<0.001*
	No	243 (81.0%)	273 (91.0%)	
Burning at the injection site	Yes	90 (30.0%)	36 (12.0%)	<0.001*
	No	210 (70.0%)	264 (88.0%)	
Flu	Yes	39 (13.0%)	21 (7.0%)	0.014*
	No	261 (87.0%)	279 (93.0%)	
Anxiety	Yes	51 (17.0%)	15 (5.0%)	<0.001*
	No	249 (83.0%)	285 (95.0%)	
Muscle pain (myalgia)	Yes	51 (17.0%)	21 (7.0%)	<0.001*
	No	249 (83.0%)	279 (93.0%)	
Fatigue	Yes	54 (18.0%)	30 (10.0%)	0.005*
	No	246 (82.0%)	270 (90.0%)	
Joint pain	Yes	54 (18.0%)	24 (8.0%)	<0.001*
	No	246 (82.0%)	276 (92.0%)	
Chills	Yes	75 (25.0%)	33 (11.0%)	<0.001*
	No	225 (75.0%)	267 (89.0%)	
Cough	Yes	45 (15.0%)	9 (3.0%)	<0.001*
	No	255 (85.0%)	291 (97.0%)	
Swelling of glands	Yes	51 (17.0%)	21 (7.0%)	<0.001*
	No	249 (83.0%)	279 (93.0%)	
Sore throat	Yes	51 (17.0%)	27 (9.0%)	0.004*
	No	249 (83.0%)	273 (91.0%)	
Shortness of breath	Yes	51 (17.0%)	15 (5.0%)	<0.001*
	No	249 (83.0%)	285 (95.0%)	
Diarrhea	Yes	27 (9.0%)	15 (5.0%)	0.055
	No	273 (91.0%)	285 (95.0%)	
Chest pain	Yes	36 (12.0%)	18 (6.0%)	0.010*
	No	264 (88.0%)	282 (94.0%)	

Similarly, following the second dose of the Sinovac vaccine, fever was the commonly reported side effect in 108 (36.0%) hypertensive participants and 57 (19.0%) non-hypertensive participants, with a significant association noticed among them (p < 0.001). Additionally, injection site pain was observed in 97 (32.3%) hypertensive participants and 41 (13.7%) non-hypertensive participants, with a significant association found among them (p < 0.001). Moreover, other side effects such as burning, swelling, and redness at the site of injection; chills; headaches; rashes; fatigue; joint pain; muscular pain; and chest pain in hypertensive and non-hypertensive participants were significantly associated (p < 0.05). On the other hand, nausea was the least reported side effect in six (2.0%) hypertensive and six (2.0%) non-hypertensive participants, with an insignificant relationship detected among them (p = 1.0), as presented in Table 3.

**Table 3:** The prevalence of side effects after receiving the second dose of the Sinovac vaccine among hypertensive and non-hypertensive participants. P-value significant at <0.05.

Variables		Hypertensive participants, n (%)	Non-hypertensive participants, n (%)	P-value
Pain at the site of injection	Yes	97 (32.3%)	41 (13.7%)	<0.001*
	No	203 (67.7%)	259 (86.3%)	
Swelling at the site of injection	Yes	84 (28.0%)	36 (12.0%)	<0.001*
	No	216 (72.0%)	264 (88.0%)	
Redness at the site of injection	Yes	30 (10.0%)	6 (2.0%)	<0.001*
	No	270 (90.0%)	294 (98.0%)	
Lymphadenopathy	Yes	51 (17.0%)	15 (5.0%)	<0.001*
	No	249 (83.0%)	285 (95.0%)	
Fever (temperature >37.8˚C)	Yes	108 (36.0%)	57 (19.0%)	<0.001*
	No	192 (64.0%)	243 (81.0%)	
Headache	Yes	45 (15.0%)	21 (7.0%)	0.002*
	No	255 (85.0%)	279 (93.0%)	
Nausea	Yes	6 (2.0%)	6 (2.0%)	1.000
	No	294 (98.0%)	294 (98.0%)	
Rashes	Yes	66 (22.0%)	24 (8.0%)	<0.001*
	No	234 (78.0%)	276 (92.0%)	
Burning at the injection site	Yes	69 (23.0%)	33 (11.0%)	<0.001*
	No	231 (77.0%)	267 (89.0%)	
Flu	Yes	39 (13.0%)	15 (5.0%)	0.001*
	No	261 (87.0%)	285 (95.0%)	
Anxiety	Yes	33 (11.0%)	15 (5.0%)	0.007*
	No	267 (89.0%)	285 (95.0%)	
Muscle pain (myalgia)	Yes	63 (21.0%)	33 (11.0%)	0.001*
	No	237 (79.0%)	267 (89.0%)	
Fatigue	Yes	60 (20.0%)	24 (8.0%)	<0.001*
	No	240 (80.0%)	276 (92.0%)	
Joint pain	Yes	54 (18.0%)	30 (10.0%)	0.005*
	No	246 (82.0%)	270 (90.0%)	
Chills	Yes	59 (19.7%)	33 (11.0%)	0.003*
	No	241 (80.3%)	267 (89.0%)	
Cough	Yes	21 (7.0%)	9 (3.0%)	0.025*
	No	279 (93.0%)	291 (97.0%)	
Swelling of glands	Yes	63 (21.0%)	21 (7.0%)	<0.001*
	No	237 (79.0%)	279 (93.0%)	
Sore throat	Yes	30 (10.0%)	18 (6.0%)	0.071
	No	270 (90.0%)	282 (94.0%)	
Shortness of breath	Yes	66 (22.0%)	18 (6.0%)	<0.001*
	No	234 (78.0%)	282 (94.0%)	
Diarrhea	Yes	27 (9.0%)	15 (5.0%)	0.055
	No	273 (91.0%)	285 (95.0%)	
Chest pain	Yes	42 (14.0%)	18 (6.0%)	0.001*
	No	258 (86.0%)	282 (94.0%)	

The satisfaction level with the Sinovac vaccine showed that most hypertensive participants (106 (35.3%)) and non-hypertensive participants (105 (35.0%)) were satisfied, and 83 (27.7%) hypertensive and 133 (44.3%) non-hypertensive participants were very satisfied with their vaccinations, while 14 (4.7%) hypertensive participants reported low levels of satisfaction, with a significant relationship seen among them (p < 0.001), as presented in Table 4.

**Table 4:** The participant’s level of satisfaction with the Sinovac vaccine. P-value significant at <0.05.

Variable		Hypertensive participants, n (%)	Non-hypertensive participants, n (%)	P-value
Overall level of participant satisfaction with the vaccine	Very Satisfied	83 (27.7%)	133 (44.3%)	<0.001*
	Satisfied	106 (35.3%)	105 (35.0%)	
	Ok	97 (32.3%)	26 (8.7%)	
	Dissatisfied	14 (4.7%)	36 (12.0%)	

## Discussion

It is necessary to provide harmless and efficient COVID-19 immunization and predict its negative effects to combat the terrible impacts of the COVID-19 epidemic on human beings. Therefore, this study demonstrated the local and systemic side effects of the Sinovac vaccine in hypertensive and non-hypertensive participants.

One observational study assessed Sinovac COVID-19 post-vaccination HRs. Most recipients were female and suffered from an underlying allergy condition. The majority of symptoms and signs were cutaneous, and they typically manifested 30 minutes after vaccination. Six patients experienced responses during the first hour [2]. These findings were identical to additional research on COVID-19 vaccinations [20]. Our study did not support the above-reported research, which indicated that HRs were not commonly observed after receiving the first and second doses of the Sinovac vaccine. Additionally, there was no past history of concomitant allergic disease in recipients.

Similarly, Riad et al. [21] studied 780 Turkish medical professionals who had just received the CoronaVac vaccine in a countrywide cross-sectional study. Side effects that appeared locally and systemically were identified four weeks after vaccination. Of these, 62.5% of the 780 recipients experienced a minimum of one side effect. The most commonly reported local adverse effect was injection site pain which was observed in 41.5% of participants, whereas tiredness (23.6%), headaches (18.7%), aches and pains in the muscles (11.2%), and pain in the joints (4.9%) were the most common systemic side effects. Significantly more female medical professionals (67.9%) than male medical professionals (51.4%) experienced local and overall side effects. Young age, past exposure to COVID-19 infection, and poor health status (chronic diseases and constant usage of drugs) were also associated with a higher incidence of CoronaVac adverse effects [21]. Our study was not consistent with the above study and revealed that fever was the most common side effect reported in both hypertensive and non-hypertensive recipients after receiving both doses of the Sinovac vaccine.

Similarly, a study conducted in Pakistan evaluated 205 participants who had received the COVID-19 vaccine. Participants in the study had an average age of 32.9 ± 7.7 years, ranging from 23 to 55. There were 117 (57.1%) women and 88 (42.9%) men. About 23 (11.2%) recipients had diabetes, 25 (12.2%) were hypertensive, and two (1.0%) had a history of asthma. Of the participants, about 40 (19.5%) had recently been diagnosed with COVID-19 infection, whereas 60 (29.3%) were actively exhibiting COVID-19 symptoms, such as sore throat and flu-like symptoms. Additionally, a clinical history of comorbidities was significantly associated with the emergence of post-vaccination adverse effects. For example, hypertension participants more frequently reported pain at the injection site (p = 0.013), migraine/headache (p < 0.001), and fatigue (p = 0.002) after vaccination [22]. These findings were corroborated by the above study showing that hypertensive participants more commonly experienced side effects than non-hypertensive participants, such as fever (p < 0.001), injection site soreness (p = 0.009), headaches (p < 0.001), and fatigue (p = 0.005) after vaccination. As far as the demographic details are concerned, the mean age of the participants was 43.42 ± 14.33 and 42.52 ± 13.32 years in hypertensive and non-hypertensive participants, respectively, with a male predominance.

In addition, a self-reporting electronic survey and a questionnaire based on 502 individuals were conducted at vaccination clinics in multiple cities around Pakistan. Participants’ ages ranged from 50.8 to 20.3 years on average. Both doses of vaccinations were given to 53% of the individuals. The most frequent symptom was injection site pain (49.8%), followed by lethargy (43.0%), muscle pain (29.5%), and edema (24.5%). After receiving the first dose of the vaccine, more severe side effects were noted, but respondents still view the vaccination favorably. Overall, 47.4% of respondents stated they were confident about the effectiveness of the vaccine, 48.6% believed that they chose to get vaccinated on their own behalf, and 79.9% said they would advise others to do the same. Additionally, the manifestation of uncommon symptoms following a vaccine was more closely associated with the existence of a comorbid condition (p < 0.001). Compared with the second dose of the vaccine, the first dose significantly increased discomfort, swelling, or soreness at the injection site (p < 0.05). More than 50% of individuals said they had symptoms such as rash, redness, tenderness, and soreness at the injection site. The most often reported symptoms of additional general problems were primarily after a second dose. These symptoms include general fatigue, muscle pain, lightheadedness, nausea/vomiting, headaches, and fever [23]. Similarly, fever was the most often reported symptom in another study, followed by dyspnea and flu-like symptoms, which contrasted with their findings [24]. Our study was not concordant with the above-mentioned studies and revealed that the mean age of the participants was 43.42 ± 14.33 and 42.52 ± 13.32 years in the hypertension and non-hypertension groups, respectively. The presence of comorbid conditions such as hypertension significantly increased the local and systemic side effects compared with participants who had no hypertension. Additionally, fever was the most commonly observed side effect after the first and second doses of the Sinovac vaccine.

There was apparently widespread reluctance to adopt the COVID-19 vaccine [25]. This trend still exists in Pakistan, where it is mostly due to sociodemographic factors, cultural and religious views, poor knowledge, and a lack of awareness and acquaintance with the functioning of vaccines [26]. It was reported by one of the studies that more than 70% of participants said they had full knowledge of the vaccine and that most of them had chosen to get vaccinated [26]. The findings of another study showed a more favorable attitude toward choosing to get vaccinated [23], which has also been noted by further investigations [27,28]. As far as our study is concerned, the majority of hypertensive patients were significantly satisfied with their vaccinations and they had no fear of side effects.

The effect of hypertension on local and systemic side effects of the COVID-19 vaccine has not been largely studied. Although it was shown that hypertension was one of the rare adverse effects of the COVID-19 vaccine [29], the incidence of minor side effects in hypertensive patients, such as fever, and pain at the injection site is unknown. The possible explanation of this finding is that hypertensive patients were generally old, with other comorbidities, such as diabetes mellitus, cardiac diseases, and low overall immunity that led to more side effects in this population. Nevertheless, the side effects of the COVID-19 vaccine noted in hypertensive patients in our study were minor and self-limiting. However, more large-scale studies are warranted to uncover the possible association of more side effects in hypertensive patients.

Limitations of the study

There are various limitations of our study. First, the study was unable to link the effectiveness of the COVID-19 vaccine with the inherent resistance that appears after COVID-19 infection because it has been demonstrated in numerous studies that the natural defense provided by COVID-19 is more protective and persists for a long time and does not have any clotting side effects. Second, the presence of side effects might have affected individuals’ willingness or capacity to contribute to the survey. Furthermore, the study may not be typical of the total population because it only included a small number of hospitals and immunization facilities in the city. Consequently, more extensive research on the widely used COVID-19 vaccinations should be carried out in this region of the world.

## Conclusions

This study concluded that the presence of hypertension significantly increased the manifestations of local and systemic side effects compared with non-hypertensive participants. Nevertheless, the side effects noted in hypertensive patients were self-limiting and minor. The higher incidence of side effects in hypertensive patients may be related to older age, low immunity, and coexisting cardiac diseases; however, more large-scale studies are needed to uncover this association. Fever, pain, and swelling at the injection site were the most commonly reported side effects after receiving the first and second doses of the Sinovac vaccine. Furthermore, participants exhibited a high level of satisfaction. To hasten vaccine acceptance and public confidence, more studies on vaccination safety are required.
